# Gefitinib radiosensitizes non-small cell lung cancer cells through inhibition of ataxia telangiectasia mutated

**DOI:** 10.1186/1476-4598-9-222

**Published:** 2010-08-23

**Authors:** Soo-Yeon Park, Young Mee Kim, Hongryull Pyo

**Affiliations:** 1 Department of Radiation Oncology, Samsung Medical Center, Sungkyunkwan University School of Medicine, 50 Irwon-dong, Gangnam-gu, Seoul, 135-710, Republic of Korea; 2 Research Institute and Hospital, National Cancer Center, 809 Madu-1-dong, ILsandong-gu, Goyang, Gyeonggi, 410-769, Republic of Korea

## Abstract

**Purpose:**

Inhibitors of epidermal growth factor receptor (EGFR) have shown dramatic results in a subset of patients with non-small cell lung cancer (NSCLC), and have also been shown to enhance the effect of ionizing radiation (IR). We investigated how gefitinib, an orally given EGFR inhibitor for NSCLC patients, can radiosensitize NSCLC cells.

**Experimental Design and Results:**

In clonogenic survival assays performed in three NSCLC cell lines, gefitinib radiosensitized NCI-H460 and VMRC-LCD but not A549 cells. Gefitinib pretreatment induced multinucleated cells after IR exposure in NCI-H460 and VMRC-LCD, but not in A549 cells. Gefitinib also inhibited activation of ataxia telangiectasia mutated (ATM) after IR-exposure in NCI-H460 and VMRC-LCD, but not in A549 cells. An ATM specific inhibitor increased IR-induced multinucleated cells in both NCI-H460 and A549 cells. Gefitinib pretreatment inhibited the gradual decrease of γH2AX foci relative to time after IR exposure in NCI-H460 but not in A549 cells. Suppression of COX-2 in A549 cells induced multinucleated cells and caused radiosensitization after gefitinib+IR treatment. In contrast, COX-2 overexpression in NCI-H460 cells attenuated the induction of multinucleation and radiosensitization after the same treatment.

**Conclusions:**

Our results suggest that gefitinib radiosensitizes NSCLC cells by inhibiting ATM activity and therefore inducing mitotic cell death, and that COX-2 overexpression in NSCLC cells inhibits this action of gefitinib.

## Background

Lung cancer is the leading cause of cancer-related deaths in men and women worldwide [1], and about 80% of lung cancers are non-small cell lung carcinoma (NSCLC). The 5-year survival rate of patients with NSCLC remains among the lowest of all major human cancers at less than 15% [2]. Obviously, novel therapeutic strategies to improve survival of patients with NSCLC are needed. Epidermal growth factor receptor (EGFR) has been regarded as an attractive target molecule for the treatment of various cancers including NSCLC. Recently developed inhibitors of this molecule have shown dramatic results in a subset of patients with NSCLC and have become a routinely applied anticancer agent for this subset of patients [3-5].

EGFR belongs to the ErbB family of plasma membrane receptor tyrosine kinases and controls many important cellular functions. Increased EGFR expression has been observed in many experimental cancer cell lines and human tumors, including NSCLC, and it has been associated with advanced tumor stage, metastasis, and poor prognosis. Previous studies have suggested that high expression of EGFR is associated with resistance to cancer therapy, including radiation therapy [6,7]. Conversely, EGFR inhibitors have been shown to enhance the effects of ionizing radiation (IR) [8-12], although the effective subset of tumors for radiosensitization by these agents has not yet been defined.

Radiation therapy remains an important part of the treatment regimen for NSCLC, especially for patients with unresectable tumors. The concurrent administration of radiation therapy and chemotherapy is the first-choice treatment option for stage III unresectable NSCLC which makes up over 30% of total NSCLC patients. However, concurrent chemo-radiation therapy is frequently toxic and a significant number of patients suffer from complications such as radiation esophagitis and radiation pneumonitis during or after this treatment [13,14]. Therefore, it may be beneficial in terms of reducing toxicity and enhancing the effect of radiation therapy if we can administer radiation therapy and EGFR inhibitors concurrently to EGFR-inhibitor-responsive patients instead of administering concurrent chemotherapy. However, the precise underlying mechanisms for the radiosensitizing effect of EGFR inhibitors remained unclear and needed to be addressed to give the basic rationale for the radiation/EGFR inhibitor combined treatment and to further enhance their effects.

In this study, we investigated how gefitinib (ZD1839, Iressa^®^), an orally given, small-molecular EGFR tyrosine kinase inhibitor that is currently used in the clinic for NSCLC patients [15], can radiosensitize NSCLC cells in order to understand its mechanism of interaction with IR.

## Results

### Gefitinib pretreatment enhances the radiosensitivity of NCI-H460 and VMRC-LCD, but not A549 cells

**Figure 1:**
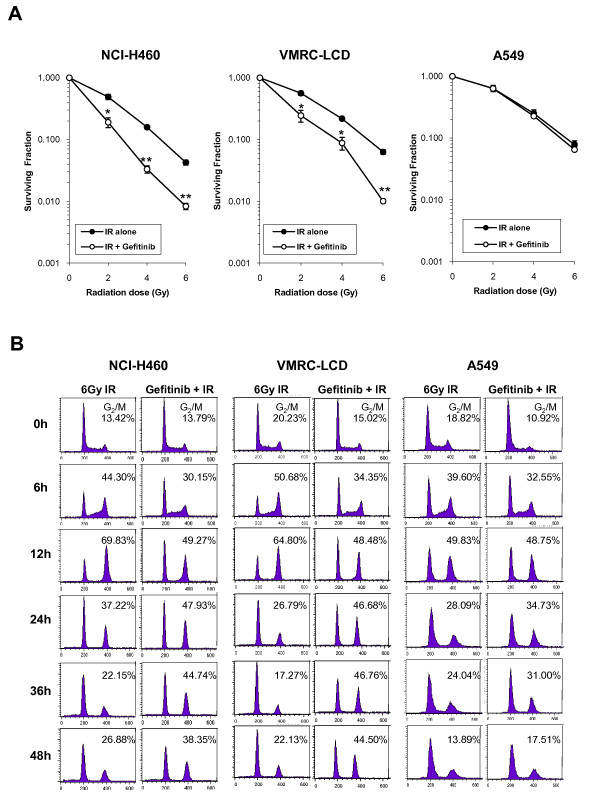
**Clonogenic cell survival and cell cycle regulation after combination treatment of gefitinib and ionizing radiation (IR) in lung cancer cells**. *A*. Clonogenic cell survival assays after gefitinib plus IR treatment. Cells were treated with gefitinib for 24 h, irradiated with graded doses of γ-radiation, incubated in the drug-containing media for another 48 h, and allowed to form colonies in drug-free medium. Surviving fractions for radiation+gefitinib were normalized by dividing by the surviving fraction for gefitinib alone. Closed circle, IR + vehicle (DMSO) treatment; Open circle, IR + 15 μmol/L gefitinib treatment. *Error Bars*, SE of three independent experiments in triplicate. **, *P *< 0.01; *, *P *< 0.05 compared with IR alone group. *B*, Cell cycle changes after gefitinib plus IR treatment. Cells were treated with 15 μmol/L gefitinib for 48 h and then irradiated. The cells were harvested after the indicated time points. Each cell cycle phase was measured by flow cytometry. Three independent experiments were performed and the results were all identical. Presented with a representative figure.

In our previous report [11], we showed that gefitinib pretreatment for 4 h enhanced the effect of IR in two NSCLC cell lines, NCI-H460 and VMRC-LCD, but not in A549 cells, also an NSCLC cell line. To further confirm the differential radiosensitizing effect of gefitinib according to cell lines, cells were exposed to 15 μmol/L gefitinib for a longer period (24 h) to allow enough time for gefitinib to take action, and then irradiated with 2, 4, or 6 Gy of IR. As shown in Figure 1A, gefitinib enhanced radiosensitivity of both NCI-H460 and VMRC-LCD cells (upper panel), and gefitinib pretreatment for 24 h was more effective than 4 h pretreatment. In contrast, gefitinib did not radiosensitize A549 cells even after prolonged preincubation with the drug (lower panel).

We previously proposed that inhibition of Chk2 phosphorylation may be an underlying mechanism for radiosensitization by gefitinib [11]. Since Chk2 is one of the core molecules in the G_2 _checkpoint which mediates IR-induced G_2 _arrest, we examined the effect of gefitinib on IR-induced G_2 _arrest. Cells were exposed to 15 μmol/L gefitinib for 48 h, irradiated with 6 Gy of γ-radiation, and then further incubated for the indicated time points. IR-induced G_2_-M arrest was decreased by gefitinib pretreatment compared to IR alone in NCI-H460 and VMRC-LCD cells at 6 or 12 h after IR exposure. However, the G_2_-M peak was not further decreased but was sustained for more than 24 h while the G_2_-M peak in IR-alone groups continued to decrease in both cell lines. In contrast, gefitinib did not affect the IR-induced G_2_-M arrest in A549 cells (Figure 1B). These results led us to hypothesize that gefitinib pretreatment attenuates IR-induced G_2 _arrest, cells subsequently enter into mitosis due to attenuation of G_2 _arrest, and the cells that entered into mitosis may be trapped in this cell cycle phase as a form of mitotic catastrophe (MC). This phenomenon was seen in NCI-H460 and VMRC-LCD but not in A549 cells.

### Gefitinib pretreatment induces multinucleated cells after IR exposure

Cancer cells severely damaged by IR are known to undergo MC or mitotic cell death (MCD) which is characterized by the formation of micronuclei and/or multinuclei [16,17]. At the same time, in mammalian cells and particularly in cancer cells, MC is mainly associated with deficiencies in cell cycle checkpoints [18,19]. The G_2_-M checkpoint is responsible for blocking mitosis in the case of damaged DNA, and inhibition or knockout of proteins that prevent premature mitosis can induce MC [20]. Therefore, we hypothesized that Chk2 inhibition and attenuation of IR-induced G_2 _arrest by gefitinib may increase IR-induced MC.

**Figure 2:**
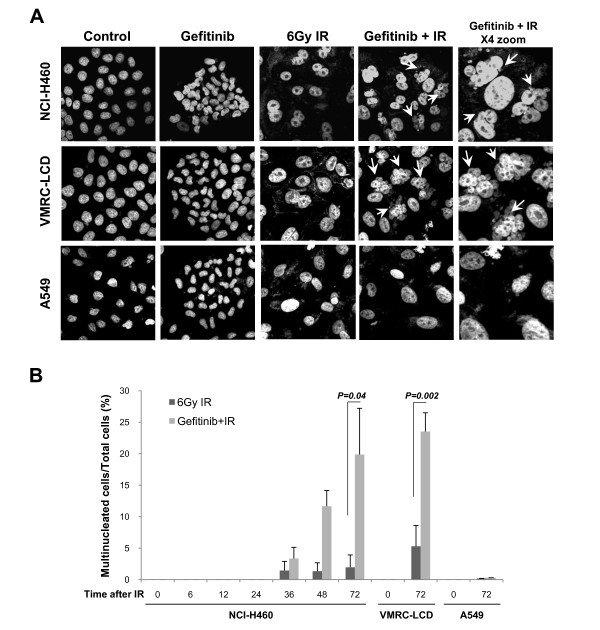
**Multinuclei formation after the treatment with gefitinib plus ionizing radiation (IR) in lung cancer cells**. *A*, Nuclear morphology of NCI-H460 (*upper*), VMRC-LCD (*middle*), and A549 (*bottom*) cells after the gefitinib plus IR treatment. Cells were grown on coverslips and treated with 15 μmol/L gefitinib for 48 h, and then irradiated with 6 Gy IR. After the indicated times, cells were fixed, stained with DAPI, and then observed under confocal microscopy (X400 magnification). Multinucleated cells were indicated with arrow. *B*, The number of multinucleated cells was counted and plotted. At least 100 cells per sample were counted. Each columns represent the means of three independent experiments; *Error bars*, SE.

To test whether gefitinib pretreatment increases MC after IR exposure, cells were stained with DAPI to visualize the nuclear morphology after gefitinib plus IR treatment. After gefitinib pretreatment followed by 6Gy IR exposure, the number of multinucleated cells increased dramatically in NCI-H460 and VMRC-LCD as compared to IR treatment alone. In contrast, multinucleated cells were not observed in A549 cells before or after the same treatment (Figure 2). These findings suggest that the enhancement of radiosensitivity by gefitinib in the tested cell lines may have occurred via MC induction. This MC may have been induced by gefitinib's inhibition of G_2 _checkpoint-related molecules including Chk2.

### IR-induced ATM and chk2 phosphorylation are inhibited by gefitinib pretreatment

**Figure 3:**
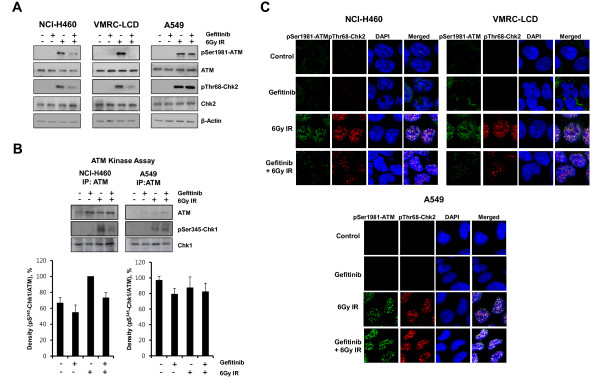
**Gefitinib inhibits ionizing radiation (IR)-induced ATM-Chk2 activation**. *A*, Western blot analyses for ATM and Chk2 phosphorylation. Cells were pretreated with gefitinib or vehicle (DMSO) for 48 h, exposed to 6 Gy IR, and were harvested and lysed for western blot analysis after 30 min. Blots representative of three independent experiments. *B*, Cells were harvested, lysed and immunoprecipitated with ATM antibody followed by in vitro kinase assays using GST-Chk1 as a substrate. The amount of phosphor-Chk1 was assessed by immunoblotting using phospho-ser345-Chk1 antibody. *C*, Cells were grown on coverslips, pretreated with gefitinib for 48 h, and then exposed to 6 Gy IR. After 30 min, cells were fixed and stained with anti-pATM and anti-pChk2 antibodies. Costaining with DAPI was done to show the nuclei. pATM (green), pChk2 (red) and DAPI (blue).

The G_2 _checkpoint is activated in response to IR-induced DNA double strand breaks, and ATM-Chk2-Cdc25C-Cdk1/Cyclin B1 is the main cascade in the G_2 _checkpoint [21,22]. Since we observed that IR-induced Chk2 phosphorylation was inhibited by gefitinib in our previous study [11], we tested whether gefitinib pretreatment also inhibits IR-induced activation of ATM (directly upstream of Chk2) as well. As shown in Figure 3A, IR-induced ATM phosphorylation was almost completely inhibited by gefitinib pretreatment in NCI-H460 and VMRC-LCD cells but not in A549 cells. Gefitinib alone did not affect ATM phosphorylation in any of the three cell lines. The status of Chk2 phosphorylation was consistent with ATM activity. ATM kinase assay showed the same results with NCI-H460 and A549 cells (Figure 3B). We confirmed these results with confocal microscopy in cells *in situ*. Phosphorylated ATM is recruited to DNA-damaged sites (foci) and participates in the DNA damage repair process [23]. As shown in Figure 3C, the number of ATM or Chk2 foci increased after IR exposure. When cells were pretreated with gefitinib, the numbers of IR-induced ATM and Chk2 foci were diminished in NCI-H460 and VMRC-LCD cells compared to those counted after IR exposure alone. However, gefitinib pretreatment did not change the number of IR-induced ATM or Chk2 foci in A549 cells. These results suggest that gefitinib pretreatment inhibits phosphorylation of ATM as well as that of Chk2 in NCI-H460 and VMRC-LCD cells, but not in A549 cells.

To test whether another EGFR inhibitor would also block IR-induced ATM/Chk2 phosphorylation, we performed the same experiments using cetuximab, which is a monoclonal antibody raised against EGFR. Cetuximab inhibited EGFR activity almost completely at the tested concentration (2 mmol/L, data not shown). In the three cell lines tested, ATM and Chk2 were phosphorylated immediately after exposure to IR. However, in contrast to gefitinib, IR-induced ATM and Chk2 phosphorylation were not inhibited when cells were pretreated with cetuximab before IR exposure (Additional file 1). This finding suggests that inhibition of IR-induced ATM-Chk2 phosphorylation is not a general characteristic of all EGFR inhibitors but a function specific to gefitinib. Therefore, this function of gefitinib may be independent of its inherent EGFR-inhibiting activity.

### Gefitinib pretreatment inhibits repair of IR-induced DNA damage

**Figure 4:**
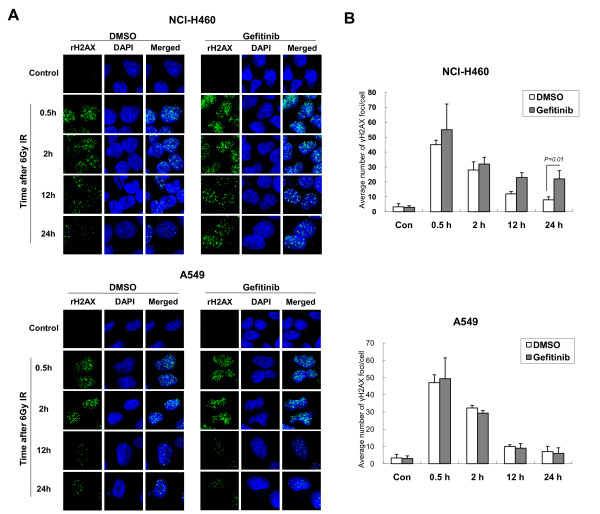
**γH2AX foci formation after combination treatment of gefitinib and ionizing radiation (IR)**. *A*, NCI-H460 and A549 cells were treated with gefitinib or vehicle for 48 h, exposed to 6 Gy IR, and fixed and stained with anti-γH2AX (green) antibody and DAPI after the indicated time points. *B*, Average number of γH2AX foci per cell was counted and plotted. At least 50 cells were counted. *Error Bars*, SE of three independent experiments.

To test the role of gefitinib pretreatment on IR-induced DNA damage, we performed immunofluorescent staining with anti-γH2AX antibody followed by confocal microscopic evaluation to observe the change in the number of γH2AX foci, a marker for DNA double strand breaks, after exposure to IR [24]. In NCI-H460 cells, γH2AX foci were induced immediately after exposure to IR (~30 min), and the number of foci decreased continuously after 12 h as a result of repair processes [25,26] in IR alone-treated groups. In contrast, the decrease in γH2AX foci according to time after IR-exposure was attenuated in the gefitinib-pretreatment groups, indicating that gefitinib inhibited the repair process of damaged DNA (Figure 4A and 4B, upper panels). In A549 cells, the pattern of induction and decrease of γH2AX foci after IR exposure was similar to that seen in NCI-H460 cells, but gefitinib pretreatment did not attenuate the decrease of γH2AX foci in A549 cells over time (Figure 4A and 4B, lower panels). Therefore, the radiosensitizing effect of gefitinib might be due to inhibition of IR-induced ATM-Chk2 activation, and this event may affect the repair process for IR-induced DNA damage in NCI-H460 or VMRC-LCD, but not in A549 cells.

### Inhibition of IR-induced ATM phosphorylation causes multinucleated cell formation in NSCLC cells

**Figure 5:**
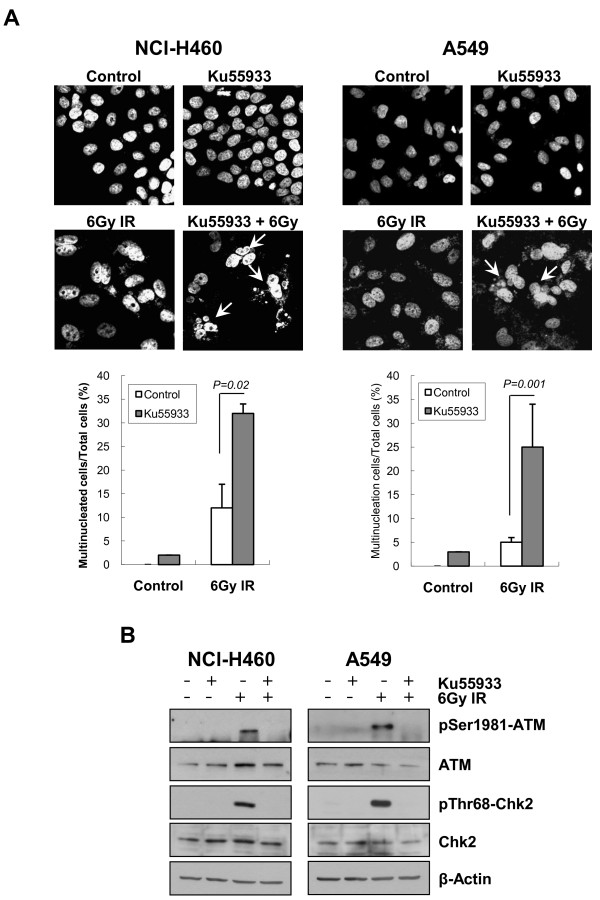
**Induction of multinucleated cells after the treatment with Ku55933, a specific ATM inhibitor**. *A*, NCI-H460 and A549 cells were grown on coverslips and treated with 10 μmol/L Ku55933 for 30 min and then exposed to 6 Gy ionizing radiation (IR). After 72 h, cells were fixed, stained with DAPI, and observed under confocal microscopy (X400). Cells with multinuclei were indicated with arrow. The number of multinucleated cells was counted and plotted. At least 100 cells were counted and each column represents the means of three independent experiments. *Error bars*, SE. *B*, Western blots after Ku55933 and IR treatment. Cells were pretreated with 10 μmol/L Ku55933 for 30 min, exposed to 6 Gy IR, and harvested after 30 min. Western blot analysis was done with anti-pATM, anti-ATM, anti-pChk2, anti-Chk2 antibodies. β-Actin was used as a loading control. Blots representative of three independent experiments.

The G_2 _checkpoint is known to play a critical role in preventing MC and the formation of multinucleated cells, as discussed previously. We performed additional experiments to determine whether inhibition of ATM phosphorylation is the cause of multinucleated cell induction in NCI-H460 cells after gefitinib plus IR treatment, and whether lack of multinucleated cell formation in A549 cells after the same treatment is due to intact IR-induced ATM phosphorylation. We administered a specific ATM inhibitor, Ku55933, with IR and analyzed the induction of multinucleated cells in NCI-H460 or A549 cells. Blocking ATM phosphorylation with Ku55933 prior to IR exposure increased the number of multinucleated cells significantly in both NCI-H460 and A549 cells (Figure 5). These results further suggest that induction of multinucleated cells by gefitinib plus IR treatment may be due to inhibition of ATM activity by gefitinib.

Collectively, gefitinib may radiosensitize NCI-H460 cells by inhibiting ATM phosphorylation and repair of damaged DNA after IR exposure, and thereby increasing multinucleated cell formation. In contrast, gefitinib may not be able to radiosensitize A549 cells because it does not inhibit IR-induced ATM phosphorylation in these cells and therefore cannot induce multinucleated cell formation and MC.

### COX-2 expression of lung cancer cells inhibits radiosensitization by gefitinib

**Figure 6:**
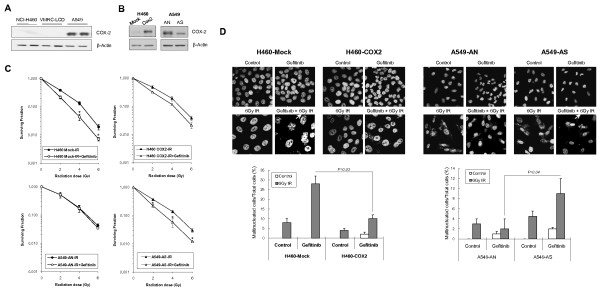
**The effect of COX-2 expression on radiosensitization by gefitinib**. Western blot analysis was done using anti-COX-2 antibody in NCI-H460, VMRC-LCD and A549 cells (*A*) or H460-Mock, -COX2, AN or AS cells (*B*). β-Actin was used as a loading control. *C*, Clonogenic cell survival assays after gefitinib plus ionizing radiation (IR) treatment in H460-Mock and -COX2, or AN and AS cells. Cells were treated with gefitinib for 24 h, irradiated, incubated in drug-containing media for another 48 h, and allowed to form colonies in drug-free medium. Surviving fractions for radiation + gefitinib were normalized by dividing by the surviving fraction for gefitinib alone. *Error Bars*, SE of three independent experiments in triplicate. *, *P *< 0.05 compared with IR alone group. *D*, Multinucleated cell induction after gefitinib plus ionizing radiation (IR) treatment in H460-Mock and -COX2, or AN and AS cells. Cells were grown on coverslips and treated with 15 μmol/L gefitinib for 48 h, and then exposed to 6 Gy IR. After 72 h, cells were fixed, stained with DAPI, and then observed under confocal microscopy (X400). Cells with multinuclei were indicated with arrow. The number of multinucleated cells was counted and plotted. At least 100 cells were counted and each column represents the means of three independent experiments. *Error bars*, SE.

We questioned why A549 cells were resistant to gefitinib's radiosensitizing function in contrast to NCI-H460 and VMRC-LCD cells. One difference between A549 and NCI-H460 or VMRC-LCD cells is the COX-2 expression level. A549 cells constitutively overexpress COX-2 while NCI-H460 and VMRC-LCD cells do not. We previously reported that COX-2-overexpressing cells showed resistance to gefitinib's antineoplastic activity [27]. Therefore, we examined the effect of COX-2 expression on gefitinib+IR-induced multinucleated cell formation. We confirmed that A549 cells express high levels of COX-2 while NCI-H460 and VMRC-LCD cells express only slight amounts of COX-2 protein (Figure 6A). We established a stably-COX-2-overexpressing cell line from NCI-H460 (H460-COX2, H460-Mock as a control cell line). We also made a COX-2-knock down A549 cell line (AS) using a COX-2-specific shRNA vector with a mock cell line as a control called AN (Figure 6B). Clonogenic assays were performed after gefitinib plus IR treatment in these COX-2-overexpressing or -suppressed cell lines. The degree of radiosensitization by gefitinib was reduced in H460-COX2 cells compared to H460-Mock cells. Conversely, gefitinib radiosensitized AS cells while causing no radiosensitization in AN cells (Figure 6C).

To further test the effect of COX-2 expression on multinucleated cell formation, the number of multinucleated cells was counted in COX-2-overexpressing or -suppressed cells after gefitinib plus IR treatment. In H460-COX2 cells, the number of gefitinib+IR-induced multinucleated cells was decreased significantly compared to H460-Mock cells (Figure 6D). On the contrary, an increased number of multinucleated cells was detected in AS cells compared to AN cells after the same treatment (Figure 6D). These results suggest that COX-2 overexpression in NSCLC cells inhibits gefitinib-induced radiosensitization. Therefore, the lack of radiosensitization of A549 cells by gefitinib seems to be due to COX-2 overexpression.

## Discussion

In the current study, we showed that gefitinib, an orally given EGFR inhibitor that is used currently to treat patients with NSCLC, can radiosensitize NSCLC cells by inhibiting ATM activity which would otherwise promote repair of damaged DNA and prevents MC after IR exposure. Gefitinib inhibited IR-induced ATM phosphorylation in the two NSCLC cell lines (NCI-H460 and VMRC-LCD) that were radiosensitized by this agent, but IR-induced ATM phosphorylation was intact after gefitinib pretreatment in an NSCLC cell line (A549) that was not radiosensitized by this drug. Gefitinib also inhibited repair of DNA double strand breaks and increased multinucleated cell formation after IR exposure in the two former cell lines while it did not in the A549 cell line. We additionally showed that an ATM-specific inhibitor, Ku55933, induced multinucleated cell formation after IR exposure in both NCI-H460 and A549 cells. These findings strongly suggest that ATM inhibition may be a primary underlying mechanism for gefitinib-mediated radiosensitization of NSCLC cells via increased MC. Therefore, gefitinib seems to act as a G_2 _checkpoint inhibitor.

The concentration of gefitinib used in the current study (15 μmol/L) is considerably higher compared to the plasma concentrations (100-500 nmole/L) that can be achieved after oral administration of gefitinib to patients. However, several pharmacokinetic studies showed extensive uptake of gefitinib into tumor in animal experiments and human investigations. Gefitinib concentrations were 42 fold higher (average tumor concentration was 16.7 μmol/L) in breast tumor and 60 fold higher (average 33.1 μmol/L) in non-small cell lung tumor than in coincident plasma samples taken from human cancer patients [28,29]. Therefore, the concentration of gefitinib we used for our experiments can be achieved in tumor of cancer patients.

After finding this novel function of gefitinib, we were curious whether this function is shared by other EGFR inhibitors, and whether it is EGFR activity-dependent. We used cetuximab, another EGFR inhibitor that is a monoclonal antibody against the ligand-binding domain of EGFR, to test whether this agent shows the same effect as gefitinib. However, cetuximab did not affect the phosphorylation level of ATM after IR exposure even though it significantly reduced EGFR activity as well as gefitinib did. Therefore, the ATM-inhibiting function of gefitinib seems to be specific to this drug. Cetuximab is also known to radiosensitize mainly head and neck cancer cells [30,31], however, the mechanism of radiosensitization may be different from that of gefitinib. We also investigated whether EGFR activation using epidermal growth factor (EGF) affects ATM phosphorylation to test for a possible connection between EGFR and ATM signaling pathways. However, we did not find elevated ATM phosphorylation after EGF administration in the tested cancer cells (data not shown). Lack of ATM-inhibiting activity by cetuximab also indicates that gefitinib's ATM-inhibiting function is independent of EGFR activity. Taken together, the ATM-inhibiting activity of gefitinib seems to be specific to this drug and it also seems to be independent of its inherent EGFR-inhibiting activity. This may be a characteristic of small molecular inhibitors that frequently targets more than one protein.

EGFR and KRAS mutations are important factors to predict response to EGFR-tyrosine kinase inhibitors [32-35]. We analyzed the status of EGFR mutations in NCI-H460, A549, and VMRC-LCD and these cells were all EGFR-wild types in exon 18,19,20, and 21 (unpublished data). In addition, according to the published data, NCI-H460 and A549 have KRAS mutations, while VMRC-LCD is a KRAS wild type [36-38]. Since gefitinib radiosensitized NCI-H460 and VMRC-LCD cells but not A549, gefitinib's radiosensitization does not seem to be related to EGFR or KRAS mutational status. A drug sensitivity and a radiosensitization by the drug seem to be mediated by quite different mechanisms.

We were curious as to why A549 cells could not be radiosensitized by gefitinib. This may be an important issue because defining the subset of tumors that respond to this drug is a necessary task to make this drug practical for radiosensitization. We found that COX-2 overexpression in A549 cells inhibited the gefitinib's MC-inducing activity. Suppression of COX-2 in A549 cells allowed for the induction of MC and radiosensitization after gefitinib plus IR treatment, while COX-2 overexpression in NCI-H460 cells reduced MC-induction and the degree of radiosensitization achieved with same treatment (Figure 6). These results show that COX-2 overexpression in NSCLC can play a critical role, although it may not be the only factor, in the development of resistance to gefitinib's radiosensitizing activity.

How COX-2 can induce this resistance to gefitinib is currently unclear. Recently, we reported that COX-2 overexpressing cancer cells upregulate ataxia telangiectasia and rad3-related (ATR) expression and activity, and that upregulated ATR induces resistance to DNA damaging agents such as IR or hydroxyurea [39]. Therefore, upregulated ATR activity in COX-2 overexpressing cancer cells may compensate for the ATM activity inhibited by gefitinib, and thereby prevent MC. On the other hand, COX-2 may directly recover gefitinib-inhibited ATM phosphorylation using as yet undefined mechanisms. Further investigation is warranted to understand the precise mechanisms involved in the resistance to gefitinib induced by COX-2.

## Conclusions

In conclusion, we propose that gefitinib radiosensitizes NSCLC cells through inhibiting IR-induced ATM activation, and therefore acts as a G_2 _checkpoint inhibitor to induce mitotic catastrophic cell death. COX-2-overexpressing cells show resistance to gefitinib's radiosensitizing activity. Our findings may contribute to the application of gefitinib or other EGFR inhibitors for combined treatment with radiation therapy in patients with NSCLC.

## Materials and methods

### Reagents

Gefitinib was provided by AstraZeneca UK Ltd. (London, United Kingdom), Cetuximab was provided by Merck (Darmstadt, Germany), Ku55933, an ATM kinase specific inhibitor, was acquired from Calbiochem (Darmstadt, Germany).

### Cell culture

Human lung large cell carcinoma cell line NCI-H460 and human lung adenocarcinoma cell line A549 were obtained from the American Type Culture Collection (ATCC, Manassas, VA, USA). Human lung adenocarcinoma cell line VMRC-LCD was obtained from the Japanese Collection of Research Bioresources (JCRB, Osaka, Japan). Cyclooxygenase(COX)-2 knocked down A549 cells by RNA interference [40] and COX-2 overexpressing NCI-H460 stable cells have been established as described previously [41]. All cells were cultured in RPMI 1640 medium supplemented with 10% fetal bovine serum (FBS, Hyclone, Logan, UT, USA), 2 mmol/L glutamine, 50 units/ml penicillin, and 50 μg/ml streptomycin (Life Technologies, Gaithersburg, MD, USA) at 37°C in an atmosphere of 5% CO_2 _and 95% air. All cell lines were qualified for mycoplasma contamination using MycoAlert^® ^Mycoplasma detection kit (Lonza, Rockland, ME, USA).

### Clonogenic assay

The effectiveness of the combined treatment with gefitinib and IR was assessed by clonogenic survival assays as described previously [40]. The surviving fraction (SF) of cells exposed to gefitinib plus IR was normalized by dividing by the SF of gefitinib alone.

### Flow cytometry

To analyze cell cycle, 1.5 ~ 3 × 10^5 ^cells were plated into 60 mm dishes for the determination of each data point. After 24 h, the cells were exposed to the appropriate concentrations of gefitinib or vehicle (DMSO) for 48 h, and then exposed to 6 Gy of γ-rays using the Gammacell 3000 Elan system (MDS Nordion Inc., Ontario, Canada). Cells were further incubated in media which contained either the drug or the vehicle for the indicated times. The cells were trypsinized (retaining all floating cells), fixed with 70% ethanol at 4°C overnight, washed with phosphate buffered saline (PBS), then incubated with 50 μg/ml of propidium iodide (PI; Sigma, St. Louis, MO, USA) and 5 μg/ml of RNase A (Amresco, Solon, OH, USA) at room temperature for 0.5 h. The number of cells at each cell cycle was evaluated with the FACS Calibur system (Becton Dickinson, San Jose, CA, USA).

### Immunofluorescence and confocal microscopy

Cells were grown on coverslips, treated with 15 μmol/L gefitinib or vehicle (DMSO) for 48 h and then exposed to 6 Gy of γ-rays. After incubation in CO_2 _incubator, cells were washed with PBS and fixed with 4% paraformaldehyde for 20 min and permeabilized in 0.5% Triton X-100 for 15 min. Anti-phosphor-ataxia telangiectasia mutated (ATM) monoclonal antibody (Rockland, Gilbertsville, PA, USA), anti-phosphor-checkpoint kinase 2 (Chk2) polyclonal antibody (Cell Signaling Technology, Beverly, MA, USA) or γ-H2AX monoclonal antibody (Millipore, Billerica, MA, USA) were diluted (1:500), and incubated with cells for overnight at 4°C. Samples were then incubated for 1 h at room temperature with Alexa 488 anti-mouse and Alexa 594 anti-rabbit secondary antibodies (Molecular Probes, Eugene, OR, USA). Nuclear staining was done with 4',6-diamidino-2-phenylindole dihydrochloride (DAPI) (Wako, Osaka, Japan). Cells were washed and mounted using mounting solution (Dako, Denmark). The images were taken with confocal microscopy (Carl Zeiss, Germany).

### Immunoblotting

Harvested cells were used for immunoblotting as described previously [40]. Equal amounts of protein were analyzed in triplicate by SDS-polyacrylamide gel electrophoresis. The following antibodies were used; anti-phosphor-Ser1981-ATM (Rockland), anti-phosphor-Thr68-Chk2 (Cell Signaling), anti-ATM (Novus, Littleton, CO, USA), anti-Chk2 (Cell Signaling) and anti-β-Actin (Sigma) antibodies. Immunoreactive proteins were detected with secondary antibodies and visualized using an enhanced chemiluminescence detection system (Amersham Bioscience, Piscataway, NJ, USA).

### In vitro kinase assay for ATM

Cells were lysed in lysis buffer (10 mM Tris-HCl pH 7.4, 1.0% Triton X-100, 0.5% Nonidet P-40, 150 mM NaCl, 20 mM sodium fluoride, 0.2 mM sodium orthovanadate, 1.0 mM EDTA, 0.2 mM PMSF). The cell lysates were centrifuged at 15,000 g for 20 min at 4°C to remove cell debris. Equal amounts of protein were incubated with anti-ATM (Novus) antibody for overnight. After addition of Protein A-agarose (Santa Cruz Biotechnology), the lysates were incubated for an additional 4 h. The beads were washed twice with the lysis beffer, once with the kinase beffer (10 mM Tris-HCl pH 7.4, 150 mM NaCl, 10 mM MgCl_2_, 0.5 mM DTT), and then incubated with kinase buffer that contains 1 mM ATP, 1 ug Chk1 kinase protein (Cell signaling) as a substrate for 20 min at 37°C. After incubation, the beads were boiled for 5 min with 5× concentrated electrophoresis sample buffer to terminate the reaction. The supernatants were separated by SDS-PAGE, and immunoblotted with ATM (Novus), Chk1, pSer345 Chk1 (Cell signaling) antibodies. Immunoreactive proteins were detected with secondary antibodies and visualized using an enhanced chemiluminescence detection system (Amersham Bioscience, Piscataway, NJ, USA).

### Statistical analysis

Statistical significance was examined using Student's *t*-tests. The two-sample *t *test was used for two-group comparisons. Values were reported as means ± standard errors (SE). *P *values < 0.05 were considered significant.

## Competing interests

The authors declare that they have no competing interests

## Authors' contributions

SYP performed research and wrote the paper, YMK analyzed data, HRP designed research and wrote the paper. All authors read and approved the final manuscript.

## Supplementary Material

Additional file 1 **Cetuximab does not inhibit ionizing radiation (IR)-induced ATM-Chk2 activation**. Western blots for ATM and Chk2 phosphorylation after IR exposure with or without cetuximab pretreatment. Cells were pretreated with cetuximab or vehicle for 48 h, exposed to 6 Gy IR, and were harvested and lysed for western blot analysis on indicated time point. Blots representative of three independent experiments. Click here for file
