# Supplementary figures and images for: Gefitinib radiosensitizes non-small cell lung cancer cells through inhibition of ataxia telangiectasia mutated
Source: Mol Cancer. 2010 Aug 23;9:222. doi: 10.1186/1476-4598-9-222 (PMC2936341; doi:10.1186/1476-4598-9-222)

## NCI-H460

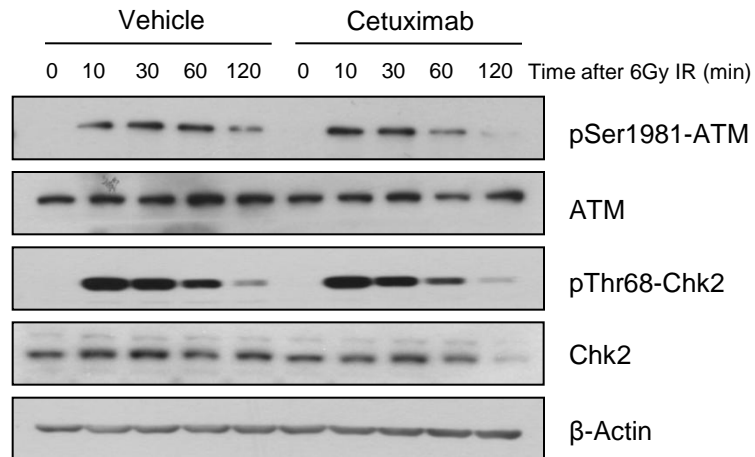

## VMRC-LCD

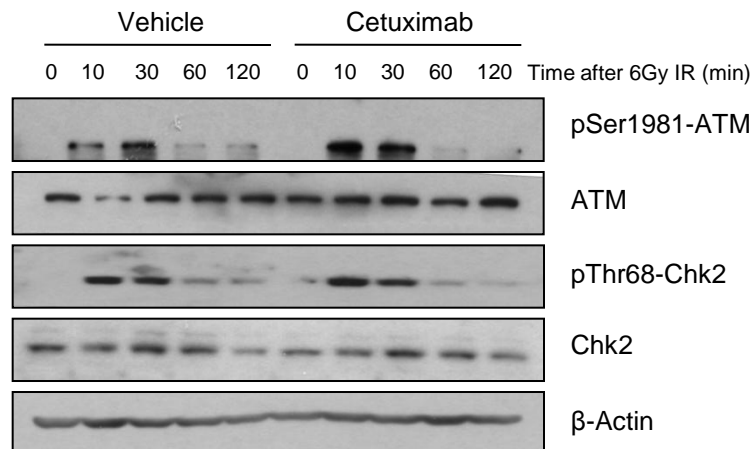

## A549

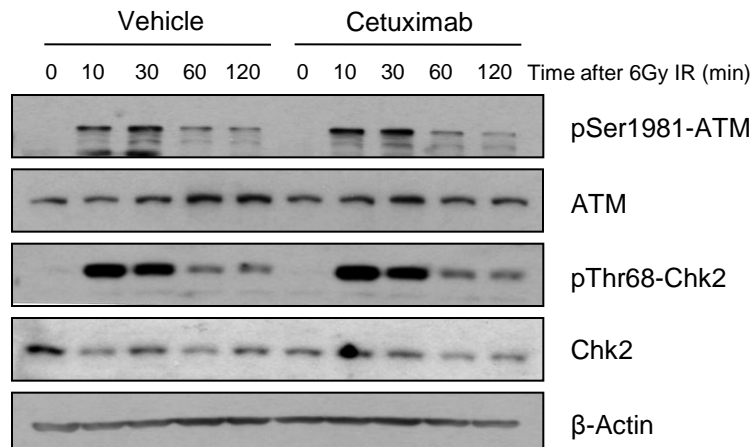

Supplement: Additional file 1 — Cetuximab does not inhibit ionizing radiation (IR)-induced ATM-Chk2 activation. Western blots for ATM and Chk2 phosphorylation after IR exposure with or without cetuximab pretreatment. Cells were pretreated with cetuximab or vehicle for 48 h, exposed to 6 Gy IR, and were harvested and lysed for western blot analysis on indicated time point. Blots representative of three independent experiments. [file 1476-4598-9-222-S1.PDF]
